# Dynamic Ocular Response to Mechanical Loading: The Role of Viscoelasticity in Energy Dissipation by the Cornea

**DOI:** 10.3390/biomimetics8010063

**Published:** 2023-02-03

**Authors:** Frederick H. Silver, Tanmay Deshmukh, Dominick Benedetto, Michael Gonzalez-Mercedes

**Affiliations:** 1 Department of Pathology and Laboratory Medicine, Robert Wood Johnson Medical School, Rutgers, The State University of New Jersey, Piscataway, NJ 08854, USA; 2 OptoVibronex, LLC, Ben Franklin Tech Partners, Bethlehem, PA 18015, USA; 3 Center For Advanced Eye Care, Vero Beach, FL 32960, USA

**Keywords:** viscoelasticity, corneal biomechanics, energy dissipation, elastic modulus, OCT, vibrational optical coherence tomography, proteoglycans, corneal diseases, loss modulus

## Abstract

We have used vibrational optical coherence tomography (VOCT) to measure the resonant frequency, elastic modulus, and loss modulus of components of the anterior segment of pig eyes in vitro. Such basic biomechanical properties of the cornea have been shown to be abnormal not only in diseases of the anterior segment but also in posterior segment diseases as well. This information is needed to better understand corneal biomechanics in health and disease and to be able to diagnose the early stages of corneal pathologies. Results of dynamic viscoelastic studies on whole pig eyes and isolated corneas indicate that at low strain rates (30 Hz or less), the viscous loss modulus is as high as 0.6 times the elastic modulus for both whole eyes and corneas. This large viscous loss is similar to that of skin, which has been hypothesized to be dependent upon the physical association of proteoglycans with collagenous fibers. The energy dissipation properties of the cornea provide a mechanism to dissipate energy associated with blunt trauma, thereby preventing delamination and failure. The cornea possesses the ability to store impact energy and transmit excess energy to the posterior segment of the eye through its serial connection to the limbus and sclera. In this manner, the viscoelastic properties of the cornea, in concert with that of the posterior segment of the pig eye, function to prevent mechanical failure of the primary focusing element of the eye. Results of resonant frequency studies suggest that the 100–120 Hz and 150–160 Hz resonant frequency peaks reside in the anterior segment of the cornea since the removal of the anterior segment of the cornea decreases the peak heights at these resonant frequencies. These results suggest that there is more than one collagen fibril network found in the anterior portion of the cornea that provides structural integrity to prevent corneal delamination and that VOCT may be useful clinically to diagnose corneal diseases.

## 1. Introduction

Energy storage, transmission, and dissipation are important functions of mammalian tissues that provide a means for locomotion, prevent mechanical failure of tissues, and up-regulate mechanotransduction [1,2,3,4,5]. Energy storage and dissipation have been addressed in several tissues, including the cornea, skin, tendon, vessel wall, and cartilage [1,2,3,4,5,6]. While it is clear that forces are generated by muscle, elastic energy is stored and transmitted via the stretching of crosslinked collagen triple helices. Energy dissipation is accomplished by the viscous sliding of collagen fibrils. The later process involves proteoglycans that reside on the collagen fibril surface [1,2,5]. However, it is not clear why dynamic forces applied to the eye do not result in corneal delamination [6]. It is also unclear whether mechanical changes to the cornea lead to changes in the retina and other visual components resulting in ocular dysfunction and disease. Results of a recent study suggest that the corneal-limbus-scleral biomechanical unit stores mechanical energy applied to the eye during loading and is postulated to transmit some of the energy through the sclera to the posterior segment of the eye [6]. The pig eye has been used as a model to study the biomechanics of the human eye. VOCT can be used to evaluate the mechanical behavior of whole eyes and excised eye components [6]. Mechanical energy storage and transmission are complex processes that involve both applied external and internal mechanical forces.

A variety of forces act on the corneal-limbus-scleral unit, including atmospheric pressure, gravity, lid pressure, intraocular pressure, muscular forces, and surface tension from the tear film [6]. These forces affect other parts of the eye and have been implicated in the development of myopia and glaucoma [7]. While the cornea is the major tissue that protects the visual components in the anterior segment of the eye from mechanical damage, it is in series mechanically with the sclera and limbus [6]. At mechanical equilibrium, all biomechanical forces must be balanced, or the sclera will continue to elongate in myopia and may affect the retina and optical nerve. Recently, several major ophthalmic conditions have been shown to be linked to corneal and scleral biomechanical properties, such as ametropia, corneal pathologies, and ocular surface disease and glaucoma [7].

A variety of methods have been proposed to evaluate corneal biomechanical behavior, as summarized in several recent publications [8,9,10,11,12,13,14,15,16]. These methods include ocular response analysis, Brillouin light scattering, Corvis ST with Scheimpflug imaging, excisional strip testing, optical coherence elastography, shear wave propagation, microindentation, and vibrational optical coherence tomography [8,9,10,11,12,13,14,15,16]. While these studies have made significant contributions to understanding changes in corneal biomechanics in health and disease, the range of reported corneal moduli is broad, from several kPa to several MPa [9]. The broad range of reported moduli [9] makes it difficult to compare the significance of different biomechanical studies. 

Both human and pig eyes have been reported to exhibit component resonant frequency peaks at about 80 Hz, 120 Hz, 150 Hz, and 250 Hz. However, the peak heights of human and pig corneas differ in magnitude [6,16]. Part of the difference can be explained by the increased stromal thickness of pig corneas compared to human eyes. Pig central corneas are about two times the thickness of the human central cornea [6,16]. The similarity of the resonant frequency peaks near 80, 120, 150, and 250 Hz of the cornea, sclera, and limbus suggest that these tissues work in concert to distribute mechanical forces and transmit energy from the front to the back of the eye [6]. Energy dissipation through the limbus and sclera may protect the cornea from changes in shape, curvature, and, therefore, refractive power [6]. However, energy dissipation through thinning (creep) of the sclera may be responsible for globe elongation observed in subjects with myopia and biophysical changes in the lamina cribrosa in glaucoma. 

The purpose of this paper is to extend previous VOCT studies on whole pig eyes and excised pig corneas to determine the relative contribution of the cornea to the viscoelastic properties of the entire eye. Since VOCT measurements have been conducted on human eyes in vivo, they can be used as a diagnostic test to study the pathogenesis of human ocular diseases [16]. 

The results of this study suggest that the viscoelastic and energy dissipation properties of isolated cornea are similar to that of whole pig eyes, suggesting that the cornea plays an important role in ocular energy dissipation. 

## 2. Materials and Methods

### 2.1. VOCT

Vibrational optical coherence tomography (VOCT) is a technology that allows one to image and measure the mechanical properties of tissues both in vivo and in vitro [6,17,18]. The technique provides a high-magnification image and measures the resonant frequency of individual macromolecular components of a tissue. The elastic modulus of each tissue component can be calculated from the resonant frequency using a calibrated empirical equation. It can be determined from a single resonant frequency measurement without assuming a value of Poisson’s ratio. It is an imaging technology that creates an image by illuminating a tissue specimen with infrared light and measuring the time delay created by the difference in the light path of the illuminating light and captured reflected light returning from the specimen. By applying an audible sinusoidal acoustic force coincident with image capture, one can measure the change in transverse displacement of the specimen as a function of frequency. With VOCT, it is possible to measure the elastic modulus (stiffness) of tissue components as described previously [6,16,17,18]. OCT image collection was accomplished using a Lumedica Spectral Domain OQ 2.0 Labscope (Lumedica Inc., Durham, NC, USA) operating in the scanning mode at a wavelength of 840 nm. The device generates a 512 × 512-pixel image with a transverse resolution of 15 μm and an A-scan rate of 13,000/s. All grayscale OCT images were color-coded to enhance the image details collected [17,18]. OCT images were used to determine the exact location of measurements made on the cornea.

### 2.2. Pig Eyes and Excised Corneas

Pig eyes were studied intact before and after part of the cornea was dissected from the entire globe. A total of 30 fresh pig eyes were obtained from Spear products (Coopersburg, PA, USA) and kept on ice during transport for testing. The pigs weighed 220 to 280 lbs and were 6 to 12 months old. Pig eyes were studied within 4 h of harvesting at Ben Franklin Tech Ventures (Bethlehem, PA, USA). It was noted that corneal thickness increased several hours after harvesting when stored on ice. 

### 2.3. Measurement of Resonant Frequency and the Elastic Modulus

The OQ Labscope was modified by adding a speaker, 2 inches in diameter (A106 Pro, JYM Electronics Co., Ltd., Shajing Town, China), to vibrate the tissue at a sound intensity of 55 dB in the VOCT studies [6,16,17,18]. The Labscope was also modified to collect and store single unprocessed raw OCT image data that was used to calculate sample displacements (deformation information) from amplitude line data. The data were processed using MATLAB software R2020a version 9.8, as discussed previously [17,18]. The mechanovibrational spectral peaks were normalized by dividing by the largest peak in each spectrum to correct for speaker orientation and varying sound levels that occur during data collection on different samples. In both the whole pig eye and the cornea studies, the sound and light were focused on the cornea.

The resonant frequency of a tissue component is defined as the frequency at which the maximum in-phase displacement is observed in the amplitude data with the applied sound wave. The measured resonant frequencies are converted into elastic modulus values using a calibration equation (Equation (1)) as reported previously [17,18]. The displacement of the tissue was measured after a transversely applied sinusoidal audible sound was applied to the specimen at frequencies ranging from 30 Hz to 300 Hz in steps of 10 Hz. Since the measurements were made at 10 Hz steps, the resonant frequencies were observed to occur at intervals of ±10 Hz. For this reason, the location of the peaks on some of the samples differ by as much as 20 Hz. The peak frequency (the resonant frequency), *f_n_*, is defined as the frequency at which the displacement is maximized after the vibrations due to the speaker are removed.

(1) $$\mathrm{Soft}\;\mathrm{Tissues}\;E\;x\;d=0.0651\;x\;\left(fn^{2}\right)+233.16$$

Since soft tissues have a density very close to 1.0, Equation (1) is valid for most tissues found in the body; where the thickness d is in m and is determined from OCT images, $fn^{2}$ is the square of the resonant frequency, and E is the elastic modulus in MPa as discussed previously [6,17,18]. Equation (1) was used to calculate the modulus values and is an empirical equation based on calibration studies using decellularized human skin (dermal collagen), human skin in vivo [5], and silicone polymers tested in both uniaxial tension and using VOCT [18]. 

Pig eye studies were conducted in vitro using a microscope stand on which the tissue to be examined was placed [6]. Whole pig eyes were examined after the fat, orbital muscles and conjunctiva were removed by dissection. The cornea was studied in the intact eye; the eye was then dissected, and the cornea was removed for VOCT measurements. A portion of the anterior cornea was removed in some samples to determine the location of the resonant frequency peaks. 

### 2.4. Statistics

All resonant frequencies and moduli were compared using an unpaired one-tailed Student’s *t*-test. All data were considered statistically different if the *p*-value was 0.05 or less.

### 2.5. Measurement of Loss Modulus

The viscous component of the modulus (loss modulus) measurement is estimated as a fraction of the elastic modulus captured at each frequency studied. To achieve this, the specimen undergoes a variation in the VOCT protocol in which the specimen is subjected to three pulses of audible sound at a known frequency from 30 to 300 Hz in steps of 10 Hz. The sample vibrational spectrum is then collected after the vibrations are terminated, and the peak heights and widths are analyzed as a function of frequency. The viscous component is obtained by dividing the change in frequency at the half height of the mechanovibrational peak, or 3 decibels down from the maximum peak in the power spectrum, by the driving frequency. This method is known as the half-height bandwidth method [5,18].

## 3. Results

**Figure 1:**
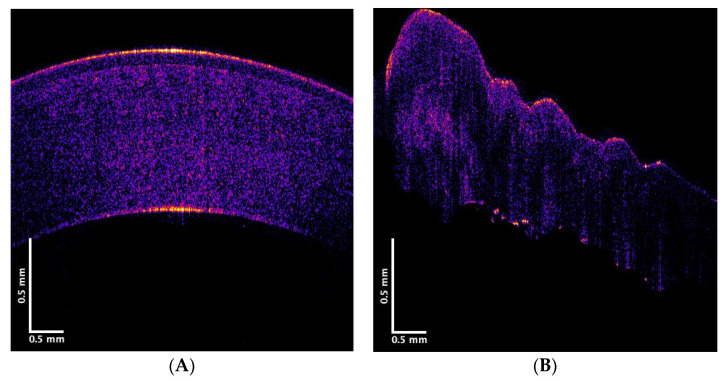
Color-coded OCT image of a normal cornea collected from a whole pig eye (**A**) and a cornea after dissection from the surrounding globe (**B**). Note the folding of the cornea after it is removed from the whole eye, indicating that it is under tension when attached to the rest of the eye.

Figure 1 displays typical OCT images of a pig cornea from a whole pig eye and from an excised pig cornea. The OCT image of the normal cornea in the whole pig eye (Figure 1A) is very similar to the image of a normal human cornea in vivo [6,16]. After dissection, and removal of the cornea, the curvature of the cornea was observed to change. The cornea becomes more convoluted and folded (Figure 1B). The increased folding of the cornea after dissection is consistent with the observation that the cornea is under tension when it is normally attached in series to the limbus and sclera. This tension is still present after the extraocular muscles are detached from the globe, suggesting that the cornea is subject to both internal and external tensile stresses in vivo.

**Figure 2:**
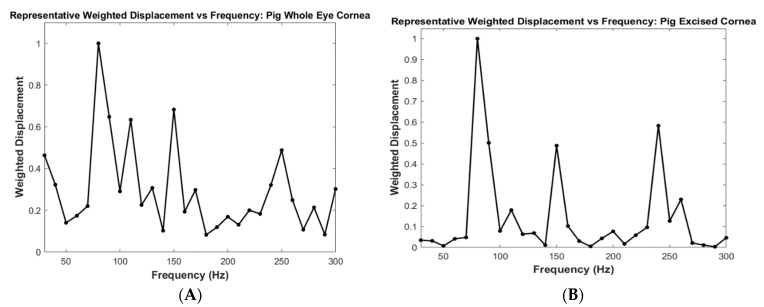
Weighted displacement versus frequency for a cornea contained in a whole pig eye (**A**) and an excised pig cornea (**B**). The prominent peaks in the excised pig eye are found at 80–90, 110–120, 150–160, and 240–250 Hz. Note that since the weighted displacement is measured at 10 Hz intervals, the resonant frequency peaks have a range of ±10 Hz.

Typical plots of weighted displacement versus frequency for the whole eye and excised cornea are shown in Figure 2. Peaks at 80–90, 100–120, 150–160, and 240–250 Hz are observed in both samples. The mechanovibrational spectrum of the excised cornea shown has fewer small background peaks. The smaller peaks present in the plot for the whole eye versus that for the excised cornea are due to vibrations from other anterior segment structures. 

**Figure 3:**
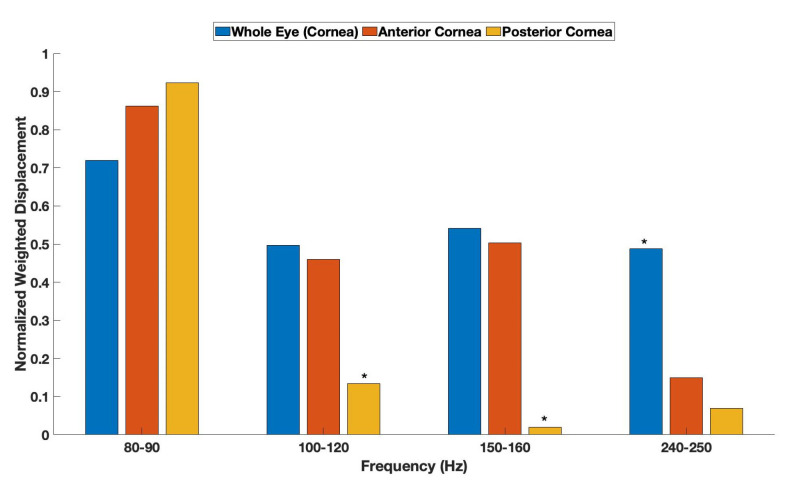
Bar graph comparing weighted displacement versus frequency for pig corneas with a thickness of 1031 ± 221 μm and excised pig corneas with a portion of the cornea removed. A comparison of normalized weighted displacement versus frequency for pig cornea in whole eye (number of samples N = 13), excised anterior portion of cornea, and the posterior part of the cornea. The average anterior cornea thickness was 760 ± 84 μm with N = 7. The posterior cornea thickness was 588 ± 218 μm with N = 10. Note the asterisks denote differences in the peak heights, as listed in Table 1. The 100–120 peak height for the posterior cornea is significantly lower than that for the whole eye and the anterior cornea. The peak height for the 150–160 peak height is significantly lower in the posterior cornea compared to the whole eye and the anterior cornea. The 240–250 peak height is higher in the whole eye than in the anterior or posterior cornea. The 240–250 Hz peak height appears to be associated with the cornea-limbus junction based on a previous study [6].

Figure 3 is a bar graph of normalized weighted displacement versus frequency comparing the mechano-vibrational peaks for pig corneas in a whole eye, an excised anterior portion of pig cornea, and an excised posterior portion of pig cornea. The results in Figure 3 suggest that the 80–90 Hz peak is associated with both the anterior and posterior segments of the whole eye, and the 100–120 peak, as well as the 150–160 Hz peak, are found primarily in the anterior portion of the cornea. The 240–250 Hz peak is mostly associated with other parts of the whole pig eye.

Table 1 shows the statistical analysis of the data shown in Figure 3. The peak heights for the 80–90 Hz peak for the anterior and posterior cornea are significantly different than that for the whole eye. The 100–120 Hz peak heights for the posterior cornea are significantly different than those from the anterior cornea and the whole eye. The peak heights for the 150–160 Hz posterior peak are significantly different from the anterior and whole eye peak heights, and the peak heights of the 240–250 Hz peak for whole eyes are significantly different than for anterior and posterior corneas.

**Figure 4:**
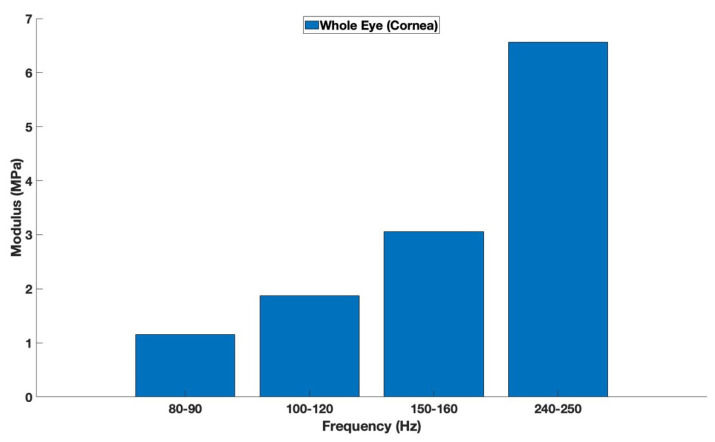
Average modulus for anterior and posterior portions of the cornea from measurements on whole pig eyes as a function of frequency (number of samples = 18). Note the average moduli for the 80–90, 100–120, 150–160, and 240–250 Hz peaks are 1.2, 1.8, 3.2, and 6.6 MPa, respectively. The average modulus for the collagenous lamellae 100–120 and 150–160 Hz peaks is 2.45 MPa.

Figure 4 is a plot of the average elastic modulus for each resonant frequency peak for the anterior and posterior portions of the cornea. The average modulus for the 80–90 Hz, 100–120 Hz, 150–160 Hz, and 240–250 Hz peaks were 1.2, 1.8, 3.1, and 6.6 MPa, respectively, for the anterior and posterior cornea. The 100–120 and 150–160 peaks appear to be collagenous tissue associated with the lamellae and circumferential collagen fibers found at the junction between the cornea and limbus. The numbers in red indicate that the differences are statistically different, with *p*-values less than 0.05, while the numbers in black are not statistically different.

**Figure 5:**
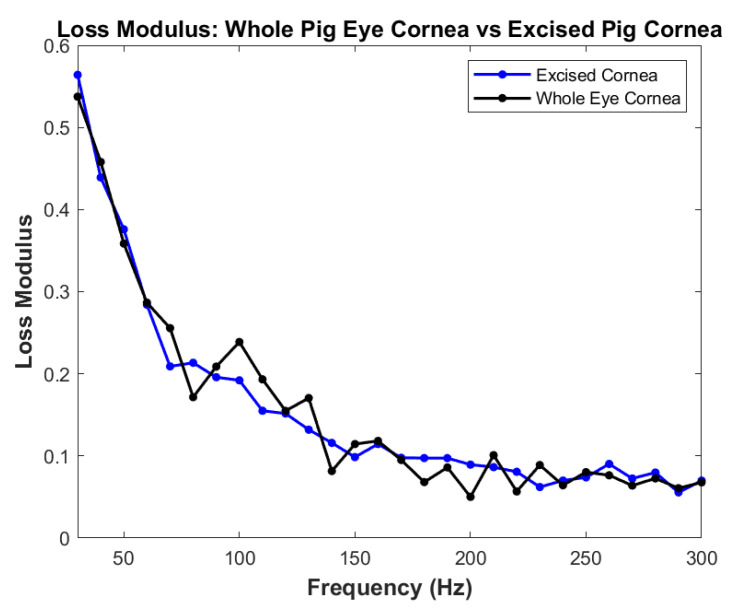
Loss modulus as a fraction of the elastic modulus determined using VOCT measurements. Note the similarity between the loss modulus for the cornea from the whole eye and the excised cornea. The high loss modulus at low frequencies suggests that the cornea acts to dissipate applied mechanical energy at low strain rates in a similar manner to the skin [5].

A plot of loss modulus as a fraction of the elastic modulus of whole pig eyes and that of the excised pig cornea is shown in Figure 5. The loss modulus is seen to be about 0.6 times the elastic modulus at low loading frequencies (30 Hz) and decreases to a fraction of about 0.1 times the elastic modulus at frequencies above 150 Hz. Note the curves overlap, and no statistical differences were observed between the whole eye and isolated pig corneas.

## 4. Discussion

### 4.1. Viscoelasticity of Cornea 

The purpose of this paper is to report the results of a VOCT study designed to evaluate the dynamic response of the pig cornea and the whole globe to the mechanical loading of the cornea. Various technologies have been used to study changes in corneal biomechanics in health and disease. These include ORA, elastography ocular response analysis, Brillouin light scattering, Corvis ST with Scheimpflug imaging, excisional strip testing, optical coherence elastography, shear wave propagation, microindentation, and vibrational optical coherence tomography [8,9,10,11,12,13,14,15,16]. Other methods have been used to study the viscoelasticity of tissues and implants [19,20,21,22,23,24,25] and of beams [26,27]. 

However, the role of viscoelasticity in corneal biomechanics has been largely ignored because of the unavailability of technology to measure the elastic and loss moduli of the cornea. The results of this study indicate that the cornea is highly viscoelastic at low strain rates (30 Hz), with the loss modulus approaching about 0.6 times the elastic modulus. This value is like that reported for human skin in vivo [5]. By comparison, decellularized human dermis, devoid of cells and proteoglycans, exhibits a loss modulus of only about 0.2 at 30 Hz [5]. Unless the strain rate dependence is considered in an experiment to measure the viscoelasticity of soft tissues, including the cornea, the value of the calculated modulus will be inaccurate by as much as a factor of 0.6. 

Our results suggest that the primary mechanical role of the cornea is to dissipate blunt impact loading applied to the outside of the eye, transmitting part of it through the series of connections to the limbus and sclera, and then finally dissipating what energy remains in the posterior segment. Transmitting impact energy to the posterior segment leads to neither corneal delamination nor tearing of the cornea, thereby maintaining its shape, transparency, and the visual acuity of the eye. 

Since the measurement of the elastic modulus depends on the strain rate, the calculation of the shear modulus will also depend on the strain rate. The cornea is 79% water [19] and will exude fluid during tensile or compressive loading, making the strain rate a significant variable, and therefore, as a result, Poisson’s ratio will be highly variable. The most common mistake in calculating the shear modulus, G, from the elastic modulus is the assumption that Poisson’s ratio is 0.5. Therefore, the expression of fluid during mechanical testing of viscoelastic tissues leads to incorrect values of both the elastic and shear moduli unless the loss modulus is also considered.

The mechanism of tensile or compression deformation of the cornea is like that of articular cartilage. During cartilage loading, the expression of synovial fluid dissipates energy applied to the cartilaginous surface. The ability to store and dissipate energy is lost in osteoarthritic cartilage when proteoglycans are removed by proteolysis and the synthesis of type I collagen replaces the type II collagen that is normally present [1]. The expression of synovial fluid by cartilage during loading and the reuptake of fluid after the load is removed via osmotic swelling of the proteoglycan molecules attached to collagen fibers is an important mechanism for energy storage, transmission, and dissipation in the musculoskeletal system [1,2,3]. The rich distribution of proteoglycans in the corneal stroma [20,28] suggests that energy dissipation in the cornea is related to proteoglycan-collagen interactions. Decorin is known to reside on the d and e bands of native collagen fibrils providing organization to the extracellular collagen fibrils, which allows fibrils to slide by each other during mechanical deformation [5]. Loss of decorin in skin, musculoskeletal, and ocular tissues is hypothesized to lead to impaired energy dissipation and irreversible sliding of collagen fibrils under normal tissue loading [1,2,3,5].

### 4.2. Young’s Modulus Versus Elastic Modulus

It is important to emphasize that Young’s modulus, or the tangent modulus (the slope of the stress–strain curve), is not equivalent to the elastic modulus, E. Young’s modulus is obtained from the slope of the stress–strain curve of the cornea. Since the cornea is non-linear and the slope increases as the fluid is rearranged during tissue stretching, the slope cannot be easily determined. Measurement of the elastic modulus can be accomplished either by construction of a stress–strain curve after all stress-relaxation processes occur in the tissue have ended, e.g., at equilibrium, via stress-relaxation or by measuring the stress or deformation in phase with applied stress [5].

### 4.3. Defining Elastic Modulus of Different Parts of the Cornea Based on VOCT Measurements

Using VOCT to measure the elastic modulus of the cornea and other anterior segment anatomical structures in the eye is made possible since the deformation of structures is measured instantaneously as the deformation occurs. The loss modulus is measured from the deformation that is out-of-phase with the applied force, which is related to the width of the mechanovibrational peak. At high frequencies, the tissue does not have time to relax, and therefore, the material becomes almost purely elastic. 

The measurements of elastic modulus of corneal and excised corneal components obtained from Figure 3 suggest that the 100–120 and 150–160 peaks are associated with the anterior portion of the cornea since the removal of the anterior layer of the cornea decreases the peak heights of these resonant frequencies. The average modulus of the 100–120 and 150–160 peaks is about 2.45 MPa, similar to what has been reported for collagen fibers from the dermis [5]. Therefore, it is concluded that the 100–120 and 150–160 Hz peaks represent the collagen fibrils of the lamellae and circumferential collagen fibers at the periphery of the cornea. 

It has been recently shown that the peaks at 100–120, 150–160, and 240–250 Hz are present not only in the cornea but also in the sclera. Since the anatomical layers of the cornea, limbus, and sclera are in series with each other, the vibrations seen in the cornea of the whole eye and the excised cornea appear to be identical. For this reason, the 240–250 Hz peak, although small in the anterior and posterior parts of the cornea, probably arises from the interface of the cornea with the limbus and limbus with sclera since previous measurements on pig corneas show this peak is present in the sclera [6]. This adds additional support for the concept that the cornea not only dissipates impact energy applied to the eye but also transfers some of the excess energy to the posterior segment through the limbus and sclera for additional dissipation. The loss of vitreous humor collagen and proteoglycans with age [29] limits the vitreous’ ability to assist with energy dissipation which may contribute to the gradual degeneration of posterior segment structures. Further viscoelastic measurements on subjects with high myopia and glaucoma are necessary to test the hypothesis that corneal and posterior segment loss of energy-dissipating properties may be associated with the development of several ocular diseases, including myopia and glaucoma.

### 4.4. Assumptions and Limitations

It is assumed in these studies that the tissue water content was unchanged during the studies and that the density of the ocular tissues studied was 1.0 g/cc and was constant. Other limitations include that the mechanical behavior was independent of the time between harvesting and testing of the pig eyes. One limitation of this study was that the ocular muscles were not present during testing, which may have an impact on the viscoelastic response measured for the whole eye.

## 5. Conclusions

Results of VOCT studies measuring the resonant frequency of porcine ocular structures suggest that the 100–120 Hz and 150–160 Hz resonant frequency peaks reside in the anterior portion of the cornea since the removal of the anterior portion of the cornea decreases the peak heights of these resonant frequency peaks.

Results of dynamic viscoelastic studies on the corneas of whole porcine eyes and isolated porcine corneas indicate that at low frequencies (30 Hz or less), the viscous loss fraction is as high as 0.6 of the elastic modulus for both the corneas of whole porcine eyes and for isolated porcine corneas. This large viscous loss is hypothesized to be associated with the proteoglycan types present and their content in the cornea. These results support the hypothesis that the cornea can dissipate blunt impact energy applied to the eye, preventing its delamination and mechanical failure. In addition, the corneal-limbus-scleral biomechanical unit assists in the transmission of excess energy applied to the cornea to the posterior segment through its series connection with the limbus and sclera. The results of this study suggest that VOCT may be a useful technique for studying changes in the in vivo viscoelasticity of the cornea and changes associated with the onset of corneal diseases.

## Figures and Tables

**Table 1:** Statistical analysis of resonant frequency data shown in Figure 3 was conducted using a one tailed unpaired Student’s *t*-test. Note the 80–90 Hz peak of the anterior and posterior cornea are significantly different than that of the whole eye. The 100–120 Hz and 150–160 Hz (150 Hz) peaks of the posterior cornea are significantly different from the whole eye and the anterior corneas. The 240–250 Hz peak of the anterior and posterior portions of the cornea is significantly different from that of the whole eye. Numbers in red are statistically significant; those in black are not.

	Anterior Cornea	Posterior Cornea
**80–90 Hz**		
**Whole Eye Cornea**	**0.03**	**0.018**
**Anterior Cornea**	**-**	**0.43**
**100–120 Hz**		
**Whole Eye Cornea**	**0.45**	**0.008**
**Anterior Cornea**	**-**	**0.009**
**150–160 Hz**		
**Whole Eye Cornea**	**0.43**	**0.004**
**Anterior Cornea**	**-**	**0.006**
**240–250 Hz**		
**Whole Eye Cornea**	**0.0016**	**0.0017**
**Anterior Cornea**	**-**	**0.06**

## Data Availability

Data are available at optovibronex.com (accessed on 26 January 2023).
